# How Should the Health Community Respond to Violent Political Conflict?

**DOI:** 10.1371/journal.pmed.0010014

**Published:** 2004-10-19

**Authors:** Anthony B Zwi

## Abstract

Violent political conflict is on the front pages, in Iraq, Afghanistan, and Sudan. This provocative piece discusses lessons we can learn from past conflicts in dealing with future ones

## Teaser

We must define better practice and promote organisational learning

Violent political conflict, and its impact, is again on the front pages—in Iraq, Afghanistan, and Sudan. While the situation in Darfur is now particularly urgent (see sidebar) [1,2,3,4,5], there are many other settings in which complex political emergencies are undermining health service provision and threatening human rights. Such emergencies have a direct impact on health (see Table 1). They also impair the functioning of health systems through, for example, destruction of infrastructure (such as clinics and vehicles), reduced access to medicines, death of health workers, and weakened national capacity for health policy-making [6].

**Table 1:**
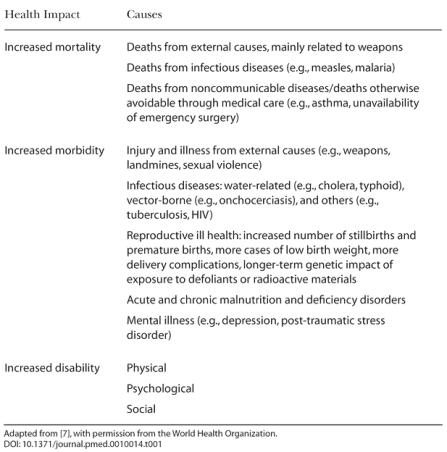
Examples of the Direct Impact of Conflict on Health

Adapted from [7], with permission from the World Health Organization

Such violent political conflicts stir us—the global health community—to discover our own humanity amidst the bloodshed. How best should we respond? Despite unique features in each setting, we must learn lessons from previous conflicts to help guide our response to current and future ones. There are six key lessons that emerge from studying health in conflict settings.

## Lessons from Conflict Settings

**Violent conflict is driven by politics and economics [7].** Complex political emergencies (1) occur within and across state boundaries, (2) have political antecedents typically relating to competition for power and resources, (3) are protracted in duration, (4) express existing social, political, economic, and cultural structures and cleavages, and (5) are often characterised by one sector preying on other parts of the community [8]. Damage to health is not just a side effect but may be the objective for violent groups. In complex political emergencies, we can typically identify three groups: the winners, the ‘conflict entrepreneurs’ (who seek the perpetuation of conflict because they profit economically or politically), and the losers, whose lives and livelihoods are imperilled. Humanitarian and relief agencies increasingly recognise that belligerents may seek to control or manipulate the inflow of humanitarian and relief resources [9]. A political economy perspective helps identify those interests, which may impede the transition to peace [7].

**Sudan—Conflict and Health**

The current crisis in Darfur reflects a devastatingly acute episode in the chronic internal conflict that has plagued Sudan since 1983. The cost of this conflict has been enormous: over 2 million lives lost, over 628,000 refugees from Sudan in neighbouring countries, and over 4 million people internally displaced [1].

In southern Sudan, the conflict has led to widespread ill health and has severely compromised the well-being of women and children. Indicators of immunisation, nutrition, primary school completion, and antenatal care are among the worst in the world. About 95,000 children under five years old died last year, most from preventable disease [2].

Statistics from UNICEF are chilling: ‘A girl born in southern Sudan has a better chance of dying in pregnancy or childbirth than of completing primary school….One in nine women dies in pregnancy or childbirth but only one in a hundred girls completes primary school’ [2].

Communities in Darfur face ongoing violence from militia supported by the government of Sudan. The fighting has resulted in large-scale destruction of villages, rape, and kidnapping. About 15,000–30,000 lives are estimated to have been lost from January 2003 to June 2004 [3]. Surveys by Médecins Sans Frontières found death rates of three to five per 10,000 people/day in Mornay and Zalinge villages (the emergency threshold level is set at one death per 10,000/day) [4]. Over 300,000 people are at risk if humanitarian access remains restricted. Of displaced Darfurians, 90% need shelter and latrines, and over half lack access to primary health care [3]. Food insecurity is widespread and is being used as a ‘weapon of war’ [5] resulting in widespread nutritional problems.

Despite widespread concern, information gaps abound, and humanitarian agencies report having access to only a fraction of those most affected. Yet, genocide is taking place in real time.

**Appreciating context is crucial.** The nature of the conflict—its background, history, and the different forms of violence involved—will greatly influence health outcomes. Most conflicts are today intra-national rather than international [10]. Internal conflicts affect populations through forced migration, violence, and human rights abuses including torture, disappearances, and rape. The forms of violence and types of health damage relate to the phase of the conflict, the sophistication of weapons used, the degree of involvement of regular military forces, the extent of terrorism employed, and the extent to which genocide is intended. Ongoing insecurity and instability may be present even after the ostensible end to the conflict, as in latter-day Afghanistan and Iraq. Challenges to governance, to service delivery, and to the reestablishment of livelihoods may persist for years. A 2003 survey in Iraq found that despite the brief duration of the war and the intent to spare hospitals and clinics from direct attack, many people suffered in the post-war period, primarily as a result of disruption to civil order [11]. Recent reports highlight the difficulties of re-establishing the health system in Iraq—partly because of a failure to appreciate the cultural and health services context [12].

**Better care can save lives.** Emergency relief efforts are increasingly based upon empirical evidence, and priority health issues are much more effectively addressed than previously. Emphasis is typically placed upon disease surveillance, immunisation, control of infectious diseases, reproductive health, water and sanitation, shelter, and nutrition [13]. Mental health, sexually transmitted infections, and HIV have recently attracted additional attention. Standards have improved, can be further improved, and warrant widespread dissemination and application. The more-established humanitarian agencies have accepted that their relief efforts must be as evidence-based as possible. This principle should also apply to the post-conflict period, during which the health of affected communities continues to suffer [14].[Fig pmed-0010014-g001]

**Figure pmed-0010014-g001:**
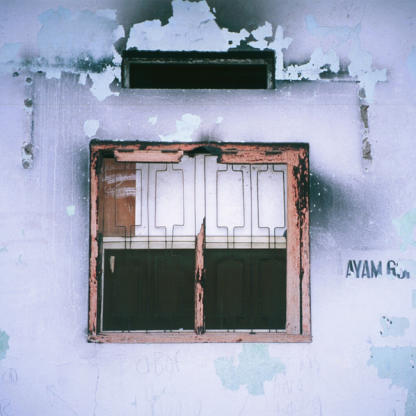
About 70% of structures were destroyed in Dili, East Timor, in the violence wrought by Indonesian militia after the referendum in 1999 (Photo: Anthony Zwi.)

**We need enhanced accountability for humanitarian action.** Despite a developing evidence base for health-related humanitarian action, evaluations of humanitarian activities have found ongoing problems. These include poor standards of delivery, duplication of efforts by different agencies, lack of coordination, and failing to learn from prior experience. The Sphere Project has advocated minimum standards for the delivery of humanitarian assistance, and has established a “Humanitarian Charter” (http://www.sphereproject.org). The project's objectives and achievements have been to improve the quality of humanitarian action and promote a movement concerned with the rights and dignity of those caught up in war and disaster [15,16]. The Active Learning Network for Accountability and Performance in Humanitarian Action (http://www.alnap.org) seeks to ensure that lessons are learned, distilled, and disseminated. At a meeting in Stockholm in June 2003, key international donors committed themselves to ‘good humanitarian donorship’, which recognises the importance of promoting standards in humanitarian action [17]. However, recent sober reflection suggests that donors and humanitarian agencies could do better: ‘An ailing humanitarian enterprise is labouring under pressures from the external environment over which it has little control, while struggling with issues internal to its own function for which it should take greater responsibility’ [18].

**Militarization of humanitarian efforts is problematic.** Multinational military forces have played a major part in recent conflicts in Kosovo, East Timor, Sierra Leone, Iraq, and Afghanistan. The military has become increasingly involved not only in waging war but also in seeking to win the peace; it is increasingly active in delivering emergency relief. It not only provides services—sometimes necessary to deliver needed relief—but also seeks to ‘win hearts and minds’ while operating within structures responsive to military and foreign policy directives. The result has seen a blurring of the separation between military and humanitarian efforts [19]. This can make humanitarian agencies a target—recent examples include the bombing of United Nations headquarters and the International Committee of the Red Cross in Iraq and the recent, reluctant withdrawal of Médecins Sans Frontières from Afghanistan following the murder of five aid workers [20]. Emerging evidence and good practice in civil-military cooperation highlights the importance of (1) promoting needs-based assistance free of discrimination, (2) civilian-military distinction in humanitarian action, (3) independence of humanitarian organisations from political pressures and interference, and (4) the security of humanitarian personnel [19].

**The transition from emergency relief to development is poorly managed.** The objectives of humanitarian relief activity (saving lives and livelihoods) differ from those of development (building sustainable systems, promoting equity, building systems of governance, and eradicating poverty). In each phase there are different actors, strategies, and approaches. The increasing politicisation of humanitarian intervention [21,22] brings threats and dangers, undermining key humanitarian principles. The balance between relief and development will vary over time and place; getting the balance right and adequately resourcing the transition warrants careful research, documentation, reflection, and the commitment of appropriate longer-term funding.

## What Gaps Remain in Our Knowledge?

Despite the knowledge we have gained on responding to violent political conflict, many important gaps remain.[Fig pmed-0010014-g002]

**Figure pmed-0010014-g002:**
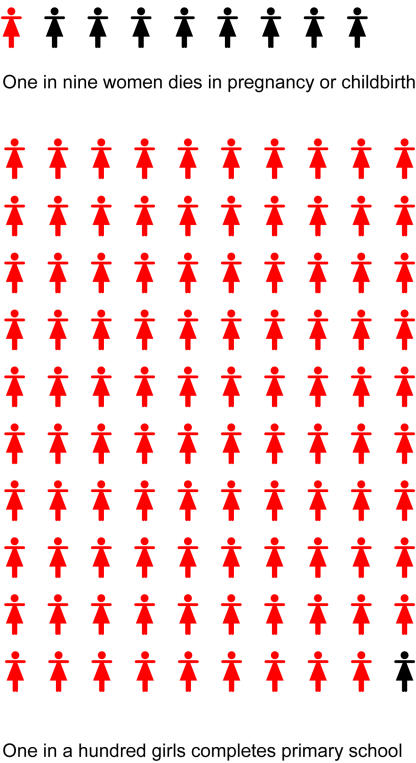
In southern Sudan, the conflict has affected the well-being of women and girls (Illustration: Margaret Shear, Public Library of Science.)

We still do not hear the voices of those most affected or of the service providers seeking to assist. The reality of people's experiences is inadequately appreciated [23]; whatever we learn of their fears, challenges, and suffering is typically represented and reported through sanitised language and media. The language used dehumanises ‘the enemy’ and blunts our senses to the reality of atrocity and to the negative effects of our own countries’ interventions. Within the health sector, ensuring that we hear the voices of service providers and carers will help bring home the reality of system disruption, destruction, and damage and will simultaneously document the mechanisms and potential for effective responses. The new communication technologies provide immense opportunity to ensure that experience is placed in the public domain from where lessons can be drawn and better practice promoted.

We know little about how communities and systems survive adversity. In most settings, the inherent ability and ingenuity of people and systems allows them to withstand instability and insecurity. Health personnel and health systems could play a valuable role in these fragile settings—assisting individuals, communities, and systems to further develop their coping strategies, adaptations, and responses. But whether health systems do so and how is unclear. Failing to support and maintain these systems may result in much greater challenges when we seek at a later stage to resuscitate them.

We also know relatively little about whether the health sector can indeed make a special contribution to building the peace. While it has been forcefully argued that the health sector is uniquely placed to play a role in peace building [24], the evidence for this remains limited [25]. We know little about how health workers see and respond to these challenging roles. The health sector could play a role in demonstrating the values and priorities of government, reflecting the relationship between those with and without resources, and the relationship between those who do and do not have protection. In the aftermath of major periods of violence, the health sector could also help to ensure that the structural inequities that preceded the violence and may have contributed to it, are not reinforced and the same injustices not recreated. But, engagement around health is not always positive: the health system is open to abuse and has been abused by repressive systems.

## From Learning Lessons to Sound Policy

Perhaps the most important gap of all is between observing lessons and putting them into practice. We urgently need to transform evidence and experience into sound policy. We need more sophisticated policy analyses, more sensitive policy-making, and more relevant research. Policy in these difficult areas will never be entirely evidence-based—often it will at best be ‘evidence-informed’. Our objective must be to promote organisations and systems that are able to reflect on experience, work with partners to critically analyse and learn, and thereby formulate better responses. Violent political conflict will continue to challenge the global health community. International policy-makers and funders must support more extensive documentation and reflection: the building blocks of better practice.
